# Laterally rotated flap for soft tissue augmentation around maxillary loaded osseointegrated dental implants: preliminary results of a pilot study

**DOI:** 10.1186/s40729-021-00376-1

**Published:** 2021-09-09

**Authors:** Jose A. Moreno Rodríguez, Julia Guerrero Gironés, Miguel R. Pecci Lloret, Antonio J. Ortiz Ruiz

**Affiliations:** 1 Private Practice, Carretera de Granada, 46. Caravaca de la Cruz, 30400 Murcia, Spain; 2 Departament of Stomatology, Faculty of Medicine, University of Murcia, Murcia, Spain

**Keywords:** Mucositis, Dental implant, Peri-implant disease, Pedicle graft, Laterally rotated flap

## Abstract

A minimal width and thickness of keratinized and attached soft tissue is desirable to prevent peri-implant diseases. This report describes the preliminary results of a pilot study of a surgical approach for soft tissue augmentation around loaded dental implants in the partially or totally edentulous maxilla. Four patients presenting eight maxillary implants with a buccal peri-implant soft tissue deficiency received a laterally rotated flap. A buccal mesial and apical recipient area was created around each implant, and a pediculated keratinized graft was rotated 90° from the distopalatal and positioned and sutured on the peri-implant buccal aspect. All implants treated showed a gain in buccal clinical peri-implant attachment (1.37 ± 0.44 mm) and buccal soft tissue levels (2.06 ± 1.40 mm) and interproximal soft tissue levels (1 ± 0.75 mm). The technique provided quality soft tissue with a gain in soft tissue thickness (3.06 ± 0.68 mm) and keratinized wide tissue (4.69 ± 0.80 mm) with minimal morbidity (1575 ± 549.67 mg of ibuprofen) and maintenance of prosthetic loading. Peri-implant soft tissue stability was maintained for 13.5 ± 1.87 months. Laterally rotated flap can be applied and provide clinical benefits to compromised implants due to the presence of buccal peri-implant soft tissue deficiency. Further studies are required to confirm these preliminary results.

## Introduction

Peri-implant mucositis is one of the most common peri-implant diseases. It was reported in more than 20% of subjects rehabilitated with dental implants [1–3]. A significant association was found between peri-implant mucositis, and smoking, implant maintenance, and peri-implant soft tissue characteristics [3].

Soft tissue quality and volume of the peri-implant mucosa are considered important factors in the prognosis of osseointegrated implants. Linkevicius et al [4] showed that if the soft tissue thickness was less than 2 mm, crestal bone loss might occur. In addition, when soft tissue width was evaluated, a wider soft tissue band was related to minimal bone remodeling [5]. A systematic review and meta-analysis by Lin et al. [6] found that a lack of keratinized tissue (KT) around osseointegrated implants was associated with plaque accumulation, peri-implant tissue inflammation, soft tissue recession, and attachment loss. Recent studies established the need for a minimal band of 2 mm of KT around osseointegrated implants and showed that a band < 2 mm was associated with more brushing discomfort, plaque accumulation, tissue inflammation, soft tissue recession, and marginal bone apical displacement and that a KT > 2 mm had a protective effect on peri-implant tissues [7–10]. Plaque accumulation and soft tissue recession have been shown even in patients with sufficient oral hygiene and regular supporting therapy [10]. Peri-implant tissue diseases have also been associated with irregular compliance when KT is lacking [9].

In peri-implant soft tissue deficiencies, soft tissue augmentation has been considered a priority, even prior to or instead of bone augmentation [11, 12]. Several surgical procedures appear in the literature to treat peri-implant soft tissue deficiencies [11–15]. Furthermore, Zucchelli et al. proposed a surgical approach for each situation in single-implant rehabilitation based on a classification of soft tissue deficiencies [16]. The prevalence of soft tissue deficiencies in no molar implants varied from 12 to 26.7% [17], and its presence is related to implant malposition, immediate implant placement, thin biotype, and other anatomical limitations [17, 18]. Furthermore, the soft tissue deficiency incidence has been reported in 57% during the first 6 months from implant loading [19].

The objective of this pilot study is to present the preliminary results of a novel surgical approach for soft tissue augmentation around loaded osseointegrated dental implants in multiple implant rehabilitations.

## Materials and methods

### Subjects and surgical site selection

A preliminary study was conducted using a laterally rotated flap. The inclusion criteria were (1) patients with partial or complete maxillary implant rehabilitation, (2) buccal soft tissue deficiency in an osseointegrated implant (lack of keratinized tissue, marginal soft tissue mobility, soft tissue width or thickness < 2 mm) [16], (3) buccal hard tissue dehiscence on the implant and buccal transparency of the underlying implant surface, (4) plaque and bleeding index of < 30%, and (5) non-plaque-retained prosthetic design; if absent, the restoration was changed to a crown with a physiologic emergence profile [20]. The exclusion criteria were (1) systemic disease that contraindicated treatment, (2) peri-implantitis disease (implant with mucositis were included in the study), and (3) smokers of more than 10 cigarettes per day. All patients were informed of the technique to be used and gave written informed consent. All clinical procedures were performed according to the Declaration of Helsinki and Good Clinical Practice Guidelines as revised in 2013. The study protocol was approved by the Research Ethics Committee of the University of Murcia (Spain) (protocol number: 2586/2019).

### Surgical procedure

**Fig. 1:**
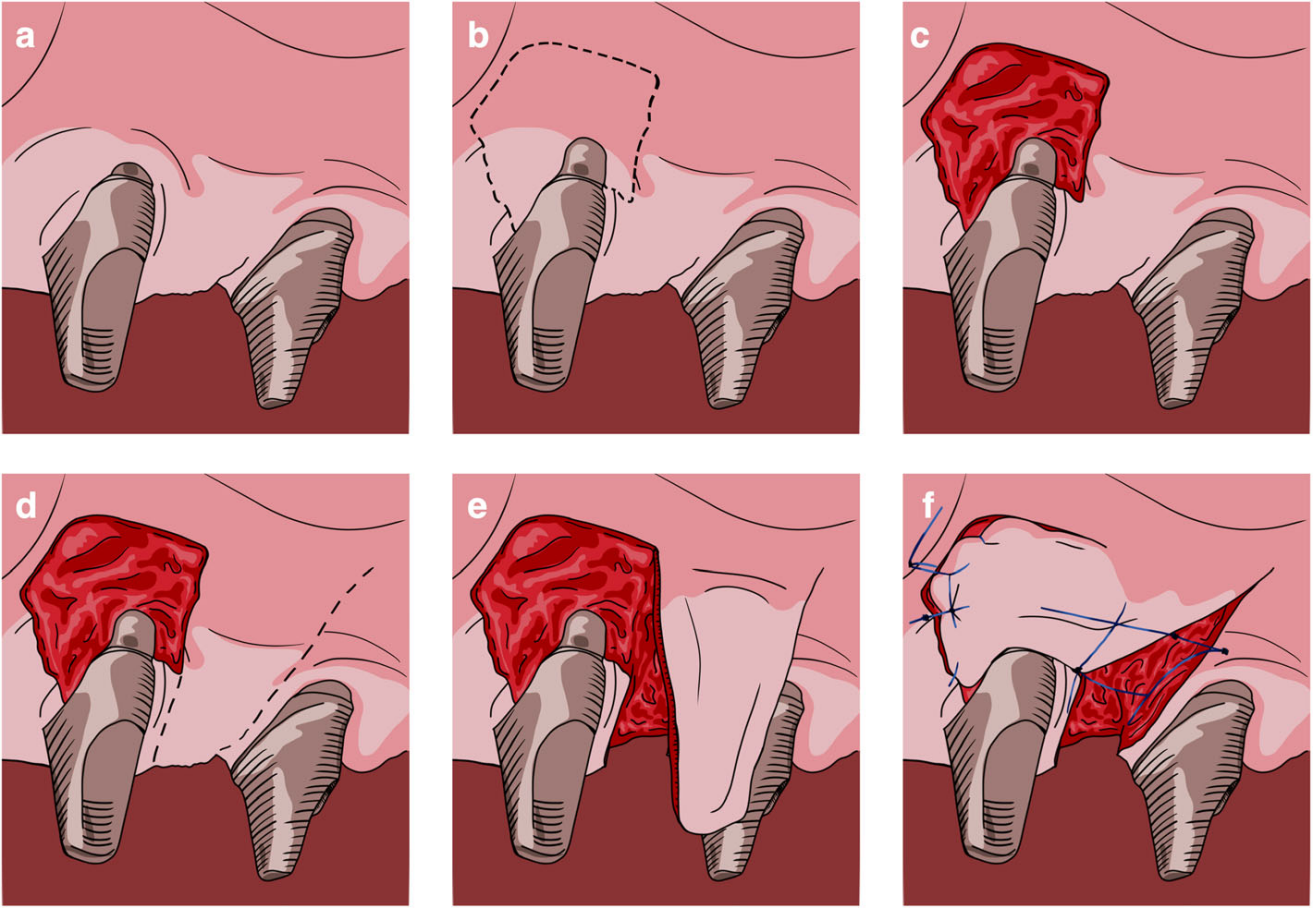
Laterally rotated flap procedure. Schematic illustrations. **a** Soft tissue deficiency around the osseointegrated dental implant. **b**, **c** The receptor area is delimited and prepared on the buccal and mesial aspects of the implant. **d** Incisions delimiting the flap to be displaced distal to the implant. **e** Keratinized pediculated flap from the distopalatal adjacent area. **f** The pediculated graft is laterally rotated 90° over the recipient site

**Fig. 2:**
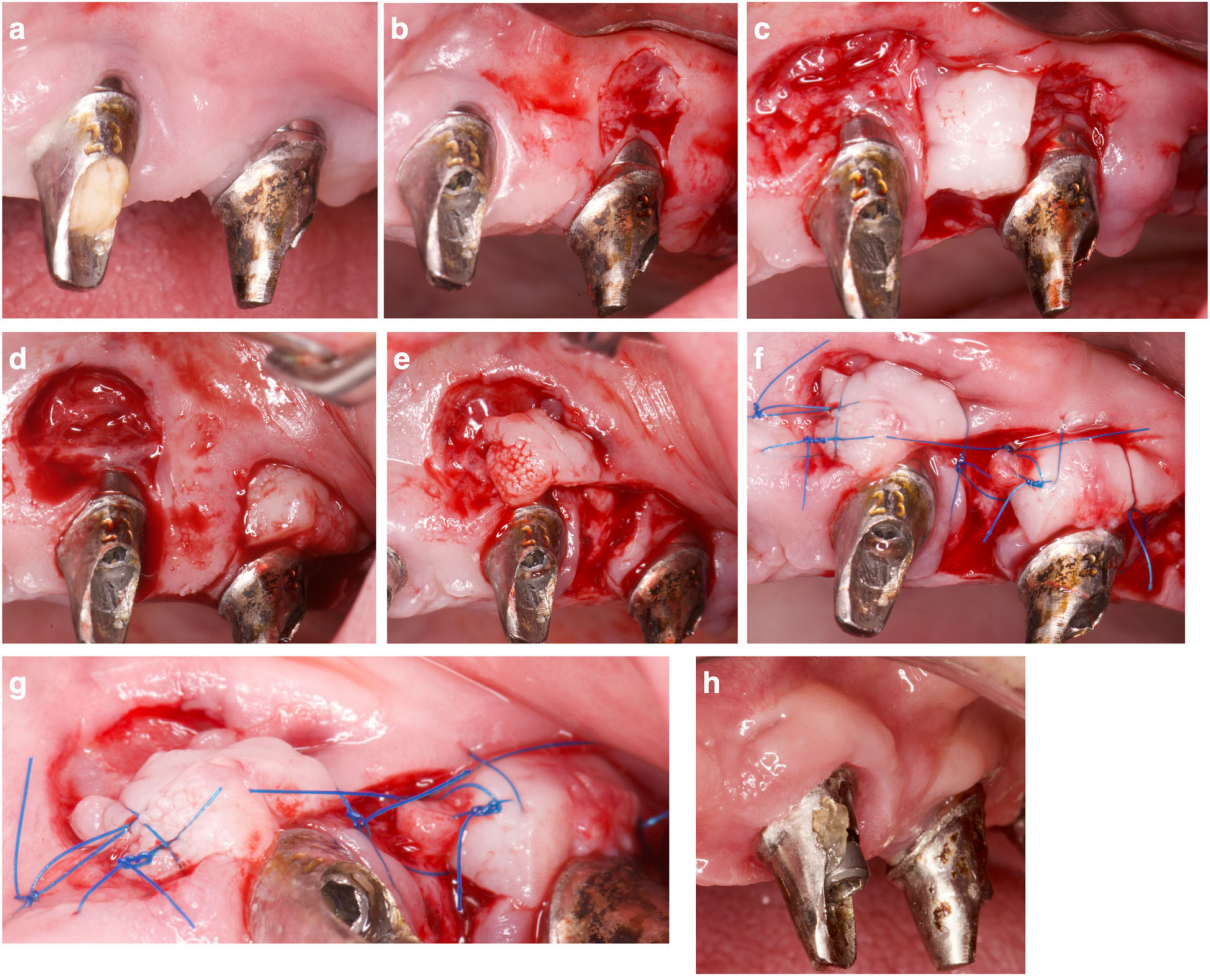
Laterally rotated flap sequence. **a** Poor peri-implant soft tissue quality and volume. **b** A pediculated graft in prepared in the distal aspect of the distal implant. The receptor area is prepared on the buccal aspect of the distal implant. **c** Recipient bleeding and firm areas in both implants and pediculated flap prepared from the distal aspect. **d** Schematic illustration. The pediculated graft is laterally rotated 90° over the recipient site. **e** Recipient bleeding and firm areas in both implants and pediculated flap prepared from the distal aspect. **f**, **g** Pediculated flaps in passive position and without tension over the recipient sites. **h** Sutures. Frontal and occlusal view. **i** Twelve months follow-up

**Fig. 3:**
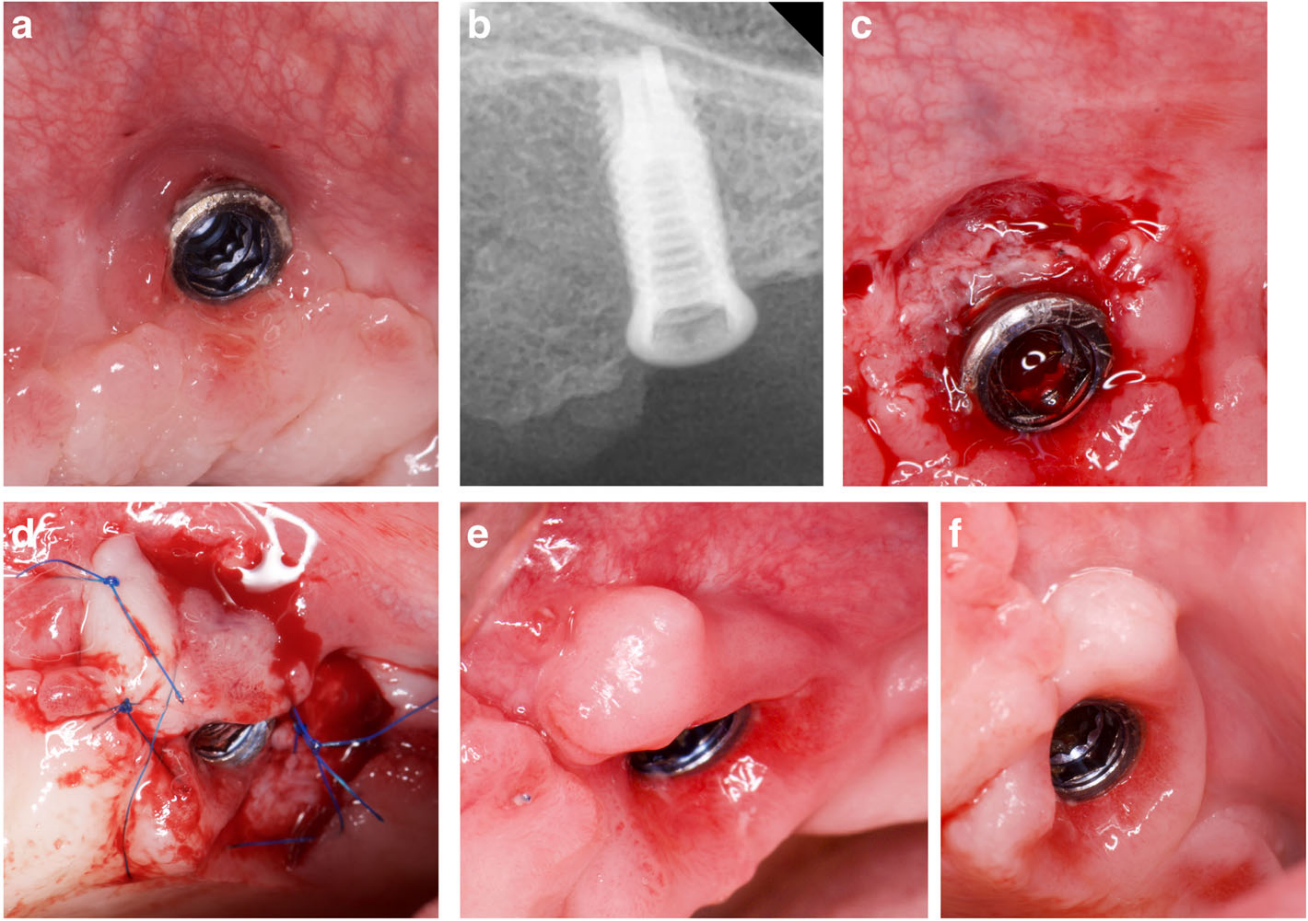
Laterally rotated flap from the distal crestal area. Maxillary dental implant in a totally edentulous arch. Dental implant buccally positioned and with 1-mm buccal surface exposure. **a** Peri-implant mucositis with lack of keratinized tissue around implant. **b** X-ray showing no peri-implantitis. **c** Preparation of the recipient site. **d** Keratinized pediculated flap laterally rotated 90° over the recipient bed and sutured. **e** Fifteen days follow-up. **f** Fifteen months follow-up, showing a large increase in soft tissue quantity and quality with optimal peri-implant tissue stability

**Fig. 4:**
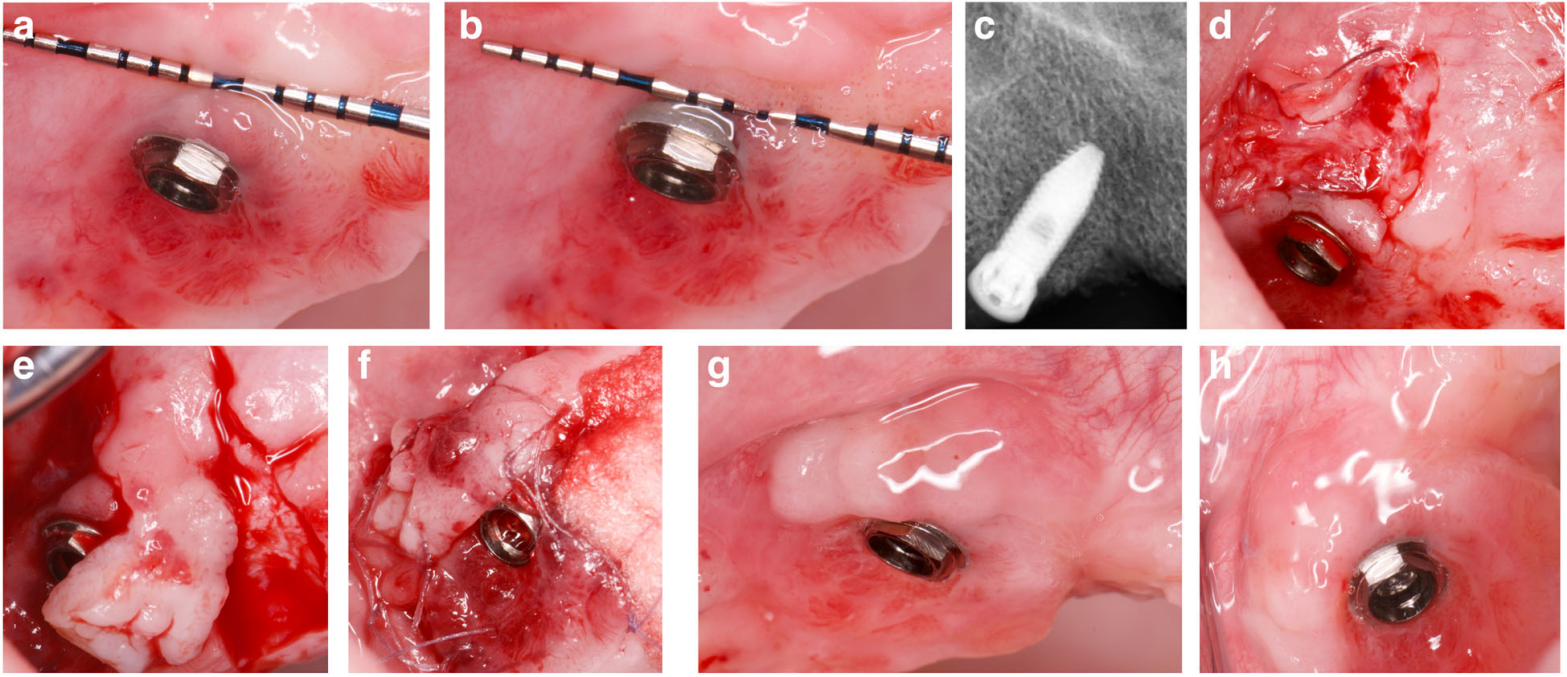
Laterally rotated flap from the distopalatal area. **a**, **b** Pre-surgical situation. Peri-implant mucositis. Lack of keratinized tissue and marginal soft tissue mobility. **c** X-ray showing no peri-implantitis. **d** Firm attached bleeding recipient area. **e** Pediculated flap from the distopalatal adjacent area. **f** Pedicle sutured to the recipient bed. Collagen sponge device protecting the donor area. **g**, **h** Nine months follow-up showing a keratinized and increased peri-implant healthy soft tissue. Frontal and occlusal view

All interventions were performed by the same surgeon (JAMR) under magnification (× 4.5). The surgical area was anesthetized with articaine/epinephrine (1:100,000) (Ultracain, Normon Laboratories S.A., Madrid, Spain). The surgical procedure followed the flap design proposed by Moreno and Caffesse in 2016 to treat soft tissue dehiscence around osseointegrated implants [21] (Figs. 1 and 2). A split-thickness recipient bed on the buccal and mesial aspects of the implant was prepared, resulting in a firm attached bleeding area. Two parallel or slightly convergent incisions were made on the distal aspect of the implant, beginning apically to the mucogingival junction and extending to the palatal tissue, where they were connected by a horizontal incision. The extension of the incisions towards the palate and the distance between them are dependent on the amount of keratinized tissue graft required for each case. Habitually, the keratinized tissue is taken from the adjacent crest; however, if greater tissue displacement is required, the incisions may be extended into the palatal tissue. The flap was prepared by partial thickness and released apically by inner superficial incision to allow passive displacement, and suturing without tension. It was latero-mesially displaced with a 90° rotation. The pedicle was sutured to the recipient bed, and the prosthetic rehabilitation re-connected (Figs. 3 and 4).

Postoperative pain and inflammation were controlled using ibuprofen (600 mg/8 h). Patients make a note of the total dose required. Patients rinsed with 0.2% chlorhexidine, twice a day for 4 weeks, without performing mechanical hygiene on the operated area. The sutures were removed 1 week later. Control visits were made at 1, 2, 3, and 4 weeks and at 3 and 6 months and 1 year, and every 6 months afterwards. At all control visits, professional maintenance of the surgical area was performed.

### Clinical parameters

The following clinical parameters were recorded before and at follow-up by the same researcher (MPL):

1. Peri-implant probing depth measured in the interproximal aspect midline and taken the high value from the soft tissue margin to the bottom of the peri-implant sulcus
2. Buccal clinical peri-implant attachment level measured from the implant platform to the bottom of the peri-implant sulcus
3. Buccal soft tissue level measured from the implant platform to the soft tissue margin
4. Buccal soft tissue thickness (measured with an endodontic file ISO15 at 2 mm from the soft tissue margin)
5. Keratinized tissue width, from the soft tissue level to the mucogingival line
6. Local bleeding score, positive or negative when bleeding or non-bleeding was elicited on probing
7. Surgical procedure morbidity based on the postoperative consumption of anti-inflammatories in milligrams of ibuprofen

### Statistical analysis

The statistical analysis was performed using R versión 4.0.3 [22]. In the descriptive analysis, values were expressed as mean ± standard deviation (SD). The Shapiro-Wilk test of the differences showed normality.

Three patients contributed to the study with several cases and one patient with one case. In order to avoid bias for that reason, we used a mixed ANOVA test to detect significant differences in clinical parameters between before surgery and after follow-up.

## Results

Four subjects were included (1 male and 3 females; age range 40–60 years). All patients were completely or partially rehabilitated with dental implants and presented buccal soft tissue deficiency in at least one maxillary implant (1 patient contributed 3 areas, 2 patients 2 areas, and 1 patient 1 area). All patients complied with revisions and maintenance instructions.

Clinical outcomes are recorded in Table 1. Before surgery, all treated areas presented bleeding on probing and < 2 mm of keratinized soft tissue thickness and width on the buccal aspect. They presented also buccal clinical attachment and soft tissue loss. All patients required changes in the prosthetic emergence profile to non-plaque-retained design. At the last follow-up visit, all cases showed no bleeding on probing, attached soft tissue, increases > 2 mm in buccal soft tissue thickness (3.06 ± 0.68 mm; *p* < 0.001) and keratinized tissue width (4.69 ± 0.80 mm; *p* = 0.008), and improvements in the buccal clinical peri-implant attachment (1.37 ± 0.44 mm; *p* = 0.008) and soft tissue (2.06 ± 1.40 mm; *p* = 0.004) levels and interproximal soft tissue (increase in peri-implant probing depth (1 ± 0.75 mm; *p* = 0.013) levels). The results remained stable for a minimum of 12 to 18 months. The anti-inflammatory consumption varied from 2 to 4 doses of ibuprofen (1575 ± 549.67 mg, 1 dose = 600 mg).

**Table 1:**
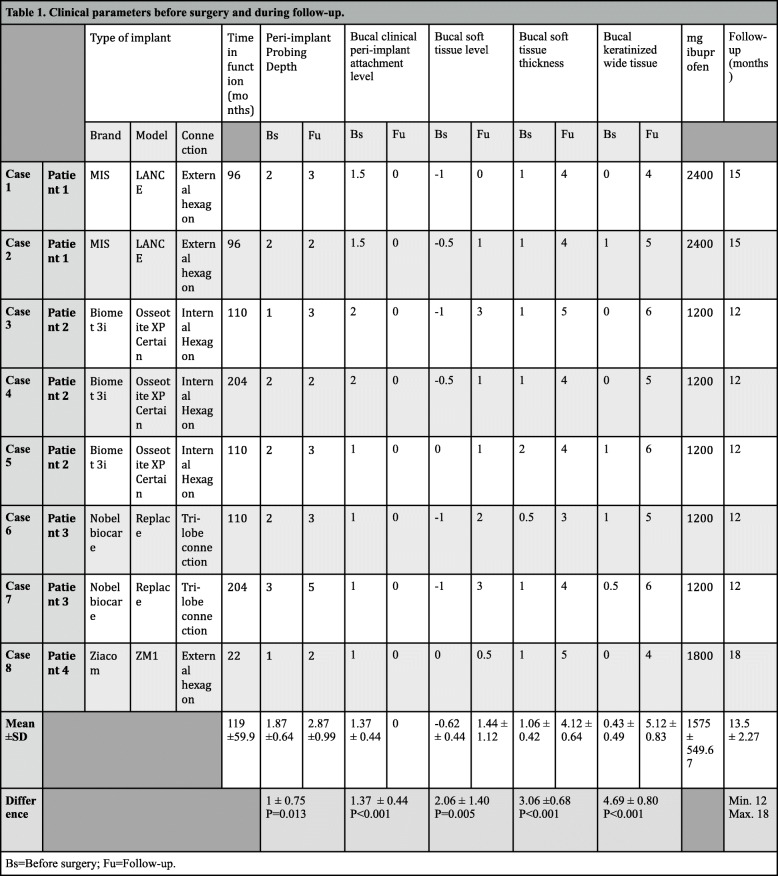
Clinical parameters before surgery and during follow-up

The mixed-ANOVA test did not detect any significant differences in the behavior of the clinical parameters of the cases as a function of the factor “cases per patient.”

## Discussion

Compromised peri-implant soft tissue represents one of the most important factors in the osseointegrated implant prognosis. Studies have underlined the importance of a minimal width and thickness of keratinized and attached soft tissue to prevent peri-implant disease [4–6, 9]. First, the lack of adequate keratinzed tissue around osseointegrated and loaded implants is associated with more plaque accumulation, tissue inflammation, mucosal recession, and loss of attachment and bone [4]; hence, the presence of < 2 mm of keratinized tissue seems to be associated with peri-implant disease in patients without adequate support performance of oral hygiene procedures around the implant [5]. Second, thin peri-implant soft tissue (< 2.5 mm) may be more prone to increase biological complications and may favor crestal bone loss [6]. Finally, peri-implant mucosa needs to depict “non-mobile” or attached tissue to facilitate transmucosal components stability of implants, prevent peri-implant inflammation and biologic complication, and preserve the marginal bone around the implant [7, 9].

Basically, two different approaches are considered for soft tissue augmentation around loaded osseointegrated implants: an apically positioned flap or a coronally advanced flap, both associated with a palatal soft tissue graft. These approaches involve two distant surgical areas, increasing the morbidity associated with graft harvesting and the subsequent healing [23, 24]. An apically positioned flap plus an epithelialized soft tissue graft has been the most common approach. However, the esthetic results [25], the high percentage of graft contraction [23, 26], the risk of wound stability failure, and graft necrosis should be considered when making decisions [27]. Bilaminar techniques associated with a connective tissue graft represent another option for soft tissue augmentation. The positioned flap may promote graft survival, while the graft may increase the stability of the displaced flap [28]. However, a lack of keratinized tissue around the implant may indicate poor tissue quality for management and displacement of a flap to cover the graft, resulting in the creation of mobile soft tissue around the implant. Furthermore, periosteal and muscular release as a step in a coronally advanced flap may result in vestibular depth reduction and greater patient discomfort [29].

In this pilot study, all cases had a mobile peri-implant soft tissue, a total lack of keratinized tissue, and < 2 mm of thickness. The soft tissue margin was at the level of or slightly apical to the implant platform. However, the described procedure results in significant improvements in peri-implant soft tissue qualities even coronal to the implant platform. The attached keratinized tissue outcomes varied from 4 to 6 mm, and the soft tissue thickness outcomes varied from 3 to 5 mm. Based on the current evidence [4–6, 9], these parameters may guarantee a good osseintegrated implant prognosis and prevent biological complications.

The laterally rotated flap has been proposed as a procedure to achieve soft tissue augmentation around implants [21] and may provide the advantages of a free keratinized graft while maintaining the increased blood supply and the stability of a pedicle flap. Furthermore, a pedicle flap results in less shrinkage over time compared with a free soft tissue graft [30], allowing an apical and buccal increase in the keratinized mucosa around the implant. Additionally, an esthetic result may be achieved when the pedicle does not need to extend to the palate. In most instances, enough keratinized tissue may be available for this approach in various specific situations. The superficial grafted tissue provides optimal connective tissue graft quality for tissue augmentation with minimal morbidity [31]. The 90° flap displacement facilitates flap mobility, without invasive muscle freedom, maintaining the disto-mesial blood supply and increasing the vestibular depth.

## Conclusion

These preliminary results suggest that the proposed surgical technique may improve soft tissue quality and quantity, in addition to the buccal transmucosal component around loaded dental implants in partial and completely edentulous patients. Randomized controlled clinical trials with medium- to long-term follow-up are needed to confirm these findings.

## Data Availability

The dataset used and analyzed during the current publication is available from the corresponding author on a reasonable request.

## Abbreviation

KT: Keratinized tissue
